# Targeting RAS guanyl releasing protein 1 promotes T lymphocytes infiltrations and improves anti‐programmed death receptor ligand 1 therapy response of triple‐negative breast cancer

**DOI:** 10.1002/ctm2.1335

**Published:** 2023-07-17

**Authors:** Xi Chen, Yuanliang Yan, Wangrui Liu, Yuanhong Liu, Abhimanyu Thakur, Qiuju Liang, Zhijie Xu

**Affiliations:** ^1^ Department of Pharmacy Xiangya Hospital, Central South University Changsha China; ^2^ Renji Hospital, Shanghai Jiao Tong University School of Medicine Shanghai China; ^3^ Pritzker School of Molecular Engineering, Ben May Department for Cancer Research University of Chicago Chicago Illinois USA; ^4^ Department of Pathology Xiangya Hospital, Central South University Changsha China; ^5^ National Clinical Research Center for Geriatric Disorders, Xiangya Hospital, Central South University Changsha China

1

Dear Editor,

Triple‐negative breast cancer (TNBC) is characterized as an aggressive form of breast cancer (BC).^1^, ^2^ Targeting immune checkpoint inhibitors (ICIs), such as programmed death receptor ligand 1 (PD‐L1), has been proposed as a potential strategy for TNBC treatment.^3^ Several monoclonal antibodies targeting PD‐L1, such as Atezolizumab, have been approved to be used for TNBC treatment.^4^ Despite ICIs show long‐term treatment effects on a faction of TNBC patients, the decreased response rates still widely exist.^5^ The intrinsic and extrinsic microenvironment heterogeneity of TNBC, including insufficient infiltration of cytotoxic T lymphocytes and immunosuppressive tumour microenvironment (TME), might drive therapeutic resistance of ICIs.^6^, ^7^ Hence, novel targets that can enhance the immunotherapeutic efficacy of TNBC need to be identified.

In the present work, we extracted three Gene Expression Omnibus (GEO) datasets regarding positive or negative PD‐L1 states in TNBC (GSE100824, GSE107764 and GSE157284). The comparisons were conducted with the cutoff of *p* < .05 and Foldchange > 1.5 (Table S1), and only one co‐upregulated gene (RAS guanyl releasing protein 1, RASGRP1) was identified, whereas no co‐downregulated genes were found (Figure 1A,B and Figure S1A–C). Validating by Spearman correlation analysis, RASGRP1 expression was positively correlated with PD‐L1 expression in GSE88847, GSE180775, and GSE58812 (Figure 1C–E). Further investigations were conducted on the clinical significance of RASGRP1 in TNBC. Through BEST database and Xiantao Tool, significantly decreased RASGRP1 expression was demonstrated in ER‐ and PR‐negative groups, as well as advanced TNM stages of BC samples (Figure S1D–I). The TISCH database further revealed that decreased RASGRP1 in malignancy cells compared to immune cells of BC samples (Figure 1F). Through Kaplan‐Meier Plotter, BC or TNBC patients with lower expression of RASGRP1, underwent unfavorable distant metastasis‐free survival, overall survival and recurrence‐free survival (Figure 1G–I and Figure S1J–L). The nomogram model further revealed that RASGRP1 could function as effective predictor of 3‐, 5‐ and 10‐ year survivals in BC patients (Figure 1J). The univariate and multivariate COX analysis also confirmed that lower expression of RASGRP1 was an independent risk factor of survivals in BC patients (Table S2).

**FIGURE 1:**
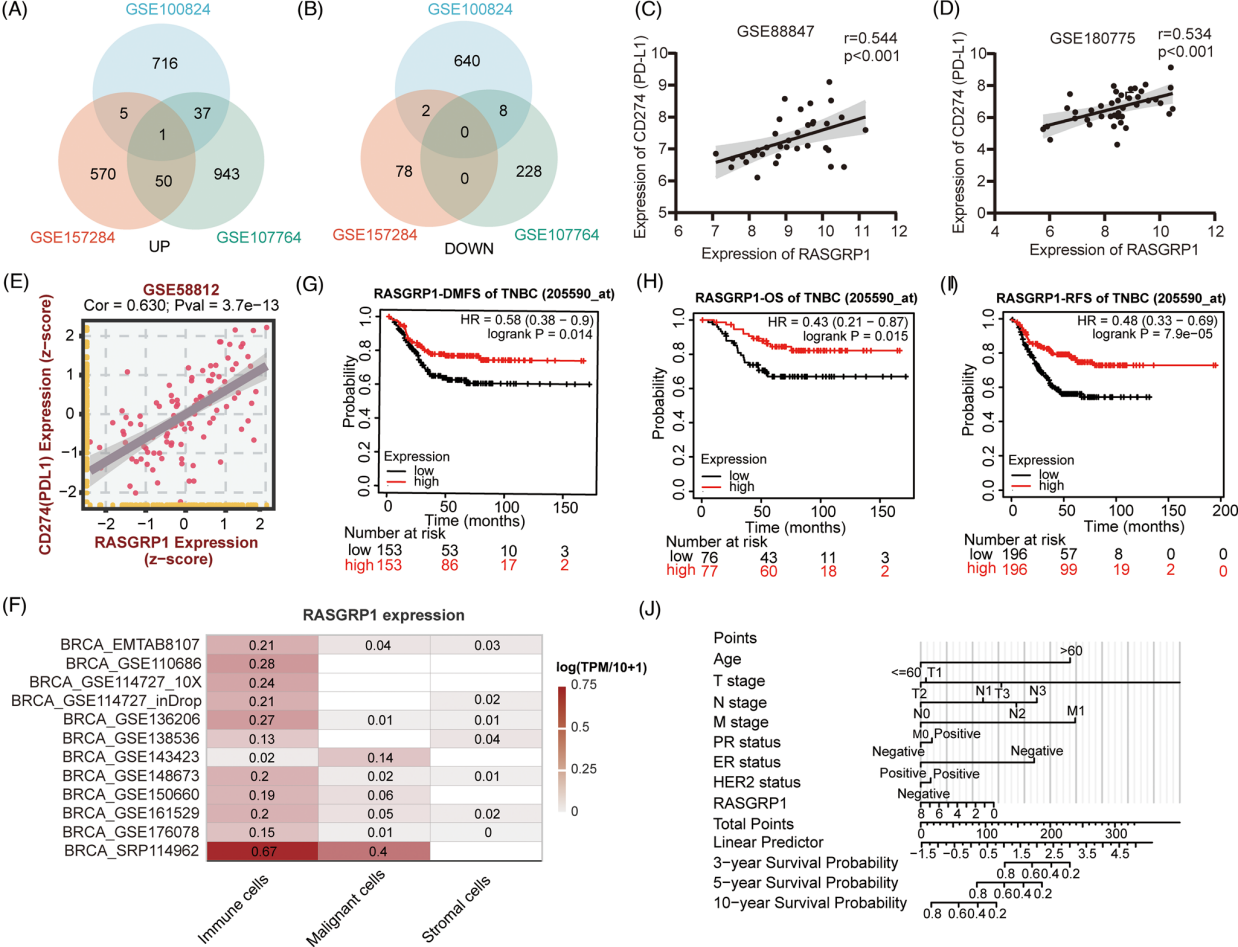
Identification of programmed death receptor ligand 1 (PD‐L1)‐associated gene RAS guanyl releasing protein 1 (RASGRP1) in triple‐negative breast cancer (TNBC). (A, B) Venn diagram to identify differentially upregulated (A) and downregulated (B) genes between high‐ and low‐expression PD‐L1 groups. (C, D) The correlation analysis between expression of RASGRP1 and PD‐L1 in GSE88847 (C) and GSE180775 (D)datasets. (E) The correlation analysis between expression of RASGRP1 and PD‐L1, performed by BEST database in GSE58812. (F) The heatmap of RASGRP1 expression in immune, malignant and stroma cells through single‐cell analysis in BC datasets from the TISCH database. (G–I) The survival curves for the distant metastasis‐free survival (DMFS) (G), overall survival (OS) (H) and recurrence‐free survival (RFS) (I) of TNBC patients in the high‐ and low‐expression groups of RASGRP1. (J) The prognostic nomogram of BC patients for the 3‐, 5‐ and 10‐year survival period is based on RASGRP1 expression levels.

To explore its biological effects, KEGG enrichment analysis revealed that RASGRP1 might be involved in the cell adhesion molecules, focal adhesion, and pathways in cancer (Figure 2A). Hence, in vitro analysis was further implemented to explore its effect. We overexpressed RASGRP1 in two human TNBC cell lines, BT549 and MDA‐MB‐231 (Figure 2B). As shown in Figure 2C–J, overexpression of RASGRP1 significantly inhibited cell viability, invasion and migration in in vitro. Furthermore, the GO and GSEA analysis manifested those genes enriched with RASGRP1 mainly involved in the immunity‐related terms, such as immune system process and T cell activation in BC (Figure 2K and Figure S2A–D). Through hallmark gene‐sets analysis, enriched pathways of RASGRP1 were associated with TNF‐α signalling in CD8^+^T cells in GSE114727_10× (Figure 2L,M) and GSE114727_inDrop (Figure 2N) in TISCH database. As a kind of cytokine, TNF‐α was reported to involve in various biological effects and promote cell proliferation, tumour recurrence, and metastasis in TNBC.^8^, ^9^ We then examined the levels of TNF‐α in vitro. Upregulation of RASGRP1 was verified to reduce TNF‐α in human BT549 and MDA‐MB‐231 cells (Figure 2O,P). Similarly, stable overexpression of RASGRP1 obviously decreased TNF‐α levels in murine 4T1 cells (Figure 2Q,R).

**FIGURE 2:**
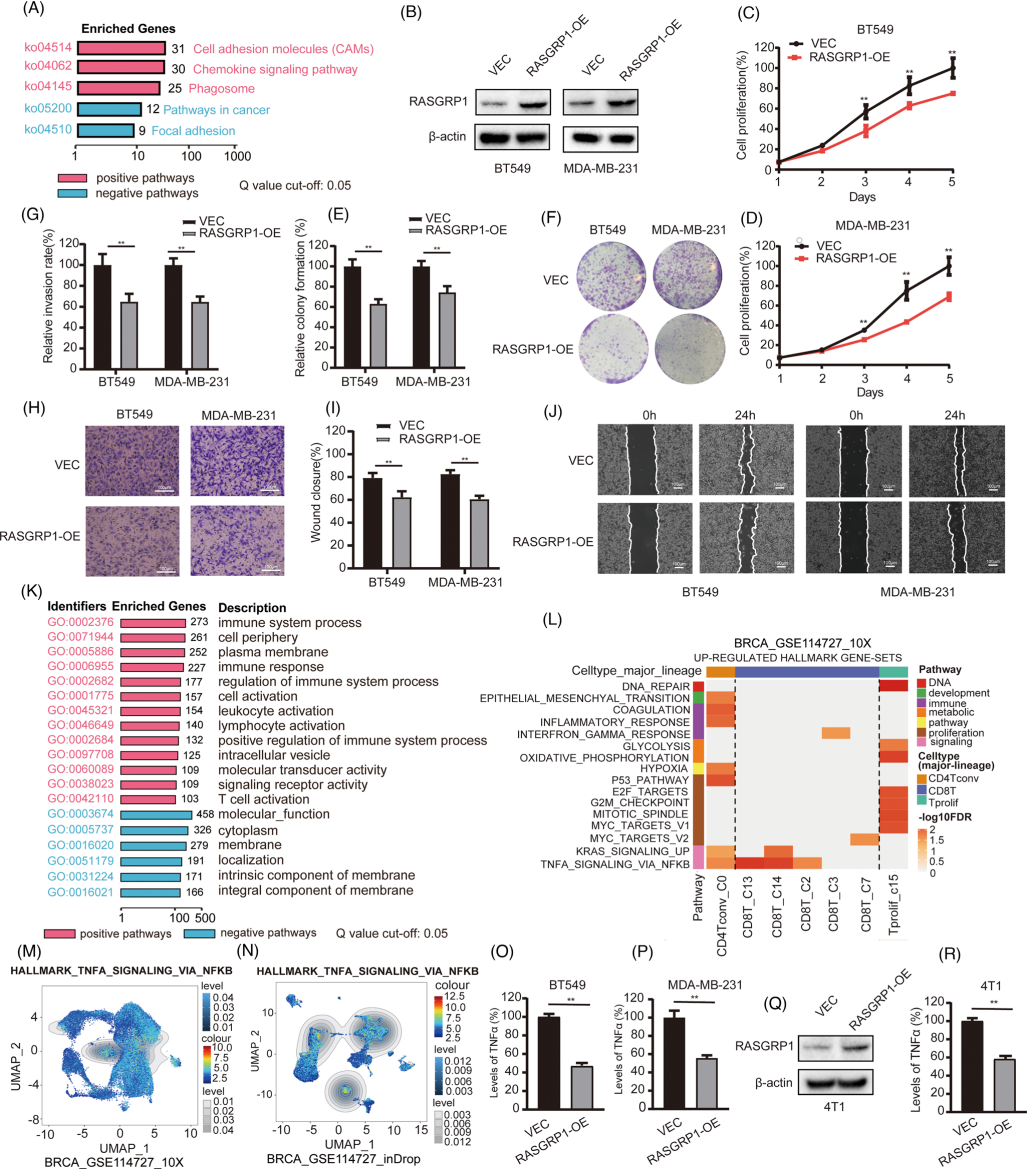
The biological and enrichment analysis of RAS guanyl releasing protein 1 (RASGRP1). (A) The KEGG pathways of RASGRP1 by BEST database. (B) After transfection of RASGRP1‐pc3.1 or vector control in BT549 and MDA‐MB‐231 cells for 48 h, the protein expression of RASGRP1 was detected by Western‐blot assay. (C, D) The cell proliferation was examined after overexpression of RASGRP1 in BT549 (C) and MDA‐MB‐231 (D) cells for 5 days. (E, F) The colony formation was analyzed in BT549 and MDA‐MB‐231 with upregulation of RASGRP1. (G, H) Examination of invasive ability in BT549 and MDA‐MB‐231 after transfection of RASGRP1‐pc3.1 using transwell assay. (I, J) The wound‐healing assays were conducted for migration analysis in BT549 and MDA‐MB‐231 cells transfected with RASGRP1‐pc3.1. (K) GO terms of RASGRP1 analyzed by the BEST database. (L) The up‐regulated hallmark gene sets of RASGRP1 in GSE114727_10×. (M, N) Enrichment scores of the hallmark TNF‐α signalling in individual cells based on GSE114727_10× (M) and GSE114727_ inDrop (N). (O, P) The levels of TNF‐α in BT549 (O) and MDA‐MB‐231 (P) after transfection of RASGRP1‐pc3.1 or vector control. (Q) The protein expression of RASGRP1 in 4T1 cells examined by Western blot assay. (R) The levels of TNF‐α in 4T1 cells. VEC: vector control; RASGRP1‐OE: RASGRP1‐overexpression.

To validate its role in TME, the BEST database and Xiantao tool showed that RASGRP1 was positively correlated with immune infiltration score and T‐lymphocyte infiltrations in BC patients (Figure 3A and Figure S2E,F). In a TNBC dataset (GSE55812), expression of RASGRP1 was positively related to CD8^+^ T cells, central memory CD8^+^ T cells, and effector memory CD8^+^ T cells (Figure 3B–E). Expression of RASGRP1 displayed positive associations with a series of T‐lymphocyte‐related immunomarkers, particularly CD8A, based on TNBC datasets (Figure 3E,F, and Figure S2G–I). Moreover, IHC staining of TNBC samples revealed that the intensity of RASGRP1 in malignancy was positively correlated with CD8 in the stroma (Figure 3G,H). Through datasets from the TISCH database, single‐cell RNA‐seq (sc‐RNAseq) analysis was further performed to verify the relationship between RASGRP1 and CD8^+^ T cell infiltrations. The sc‐RNAseq analysis showed that RASGRP1 were mainly distributed in CD8^+^ T cells surrounding TME of BC (Figure 3I–K and Figure S3A–Q). Additionally, sc‐RNAseq analysis was conducted to testify to the expression of RASGRP1 in TNBC samples based on EMTAB8107 and GSE136206. As shown in Figure 3L–T, samples with higher expression of RASGRP1 in malignant cells, showed a relatively higher abundance of CD8^+^ T cells in stroma cells. These data suggested that RASGRP1 might be relevant to the infiltrations of CD8^+^ T cells in TNBC.

**FIGURE 3:**
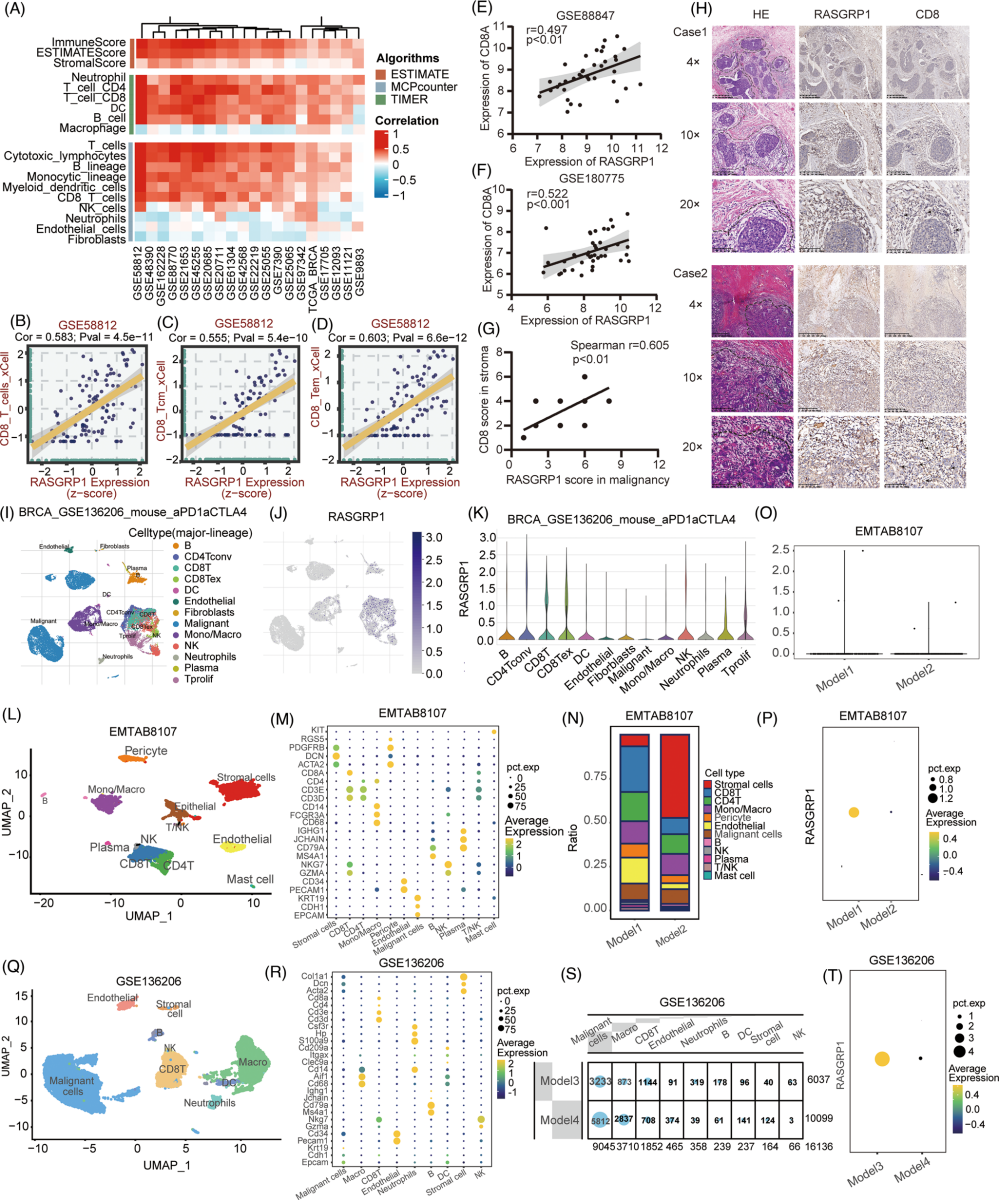
The significance of RAS guanyl releasing protein 1 (RASGRP1) in immune cell infiltration of BC. (A) The heatmap was plotted for the association between immune cells infiltration and RASGRP1 expression, using ESTIMATE, MCP counter and TIMER algorithm based on GEO datasets. (B–D) The correlation between RASGPR1 and CD8^+^ T (B), CD8^+^ Tcm (C) and CD8^+^ Tem cells (D) through the xCell algorithm in GSE58812. (E, F) The association of RASGRP1 with CD8A in GSE88847 (E) and GSE180775 (F). (G, H) The intensity of RASGRP1 and CD8 in triple‐negative breast cancer (TNBC) specimens through IHC assays. Dashed lines outline the edge of tumour (T) and stroma (S) zones. The black arrow mark denotes a highly positive CD8+ representative area. (I, J) Uniform manifold approximation and projection (UMAP) plots for major‐lineage cell types (I) and RASGRP1 expression (J) surrounding BC microenvironment in GSE136206. (K) The violin plot revealing RASGRP1 expression in major‐lineage levels in GSE136206. (L, Q) UMAP plots showing the integration of total cell types in EMTAB8107 (L) and GSE136206 (Q). (M, R) The dot plot showing the expression of indicated genes in cell clusters in EMTAB8107 (M) and GSE136206 (R). (N) The ratio of cell‐type distribution between Model1 and Model2 in EMTAB8107. Model1: human Her2‐positive samples; Model2: human TNBC samples. (O, P) The violin plot (O) and dot plot (P) showing the expression of RASGRP1 in Model1 and Model2 using EMTAB8107. (S) The cell counts of clusters in Model3 and Model4 using GSE136206. Model3: KPB25Luv−Nottreated cells (TNBC mouse cells from a K14‐Cre; Tp53f/f; Brca1f/f tumour undergoing UV without ICIs treatment); Model4: 11−Apobec−Not treated cells (mouse cells from Tp53^−/−^ and Apobec3 overexpressed syngeneic transplant tumour of TNBC without ICIs treatment). (T) The dot plot illustrating the expression of RASGRP1 in Model3 and Model4 using GSE136206. CD8Tcm: central memory CD8^+^ T cells; CD8Tem: effector memory CD8^+^ T cells.

We further investigated whether RASGRP1 could affect anti‐PD‐L1 response in TNBC. Through the TIDE algorithm, the scores predicted unfavourable ICI responses were decreased in the high RASGRP1 group (Figure S4). Subsequently, an orthotopic transplantation in vivo mice model was conducted to confirm its function. The BALB/c mice, bearing xenograft tumours of 4T1 cells with vector or stably overexpressed RASGRP1, were treated with PBS or anti‐PD‐L1 agent (Atezolizumab) (Figure 4A). As shown in Figure 4B–E, overexpression of RASGRP1 led to lower tumour weight and volume, while no change was found in body weight. The above results suggested that RASGRP1 in tumour cells may be correlated with infiltrations of CD8^+^T cells, which act as a predominant factor for ICI responses.^10^ Hence, we explored the proportion of CD8^+^ T cells from peripheral blood in each group. As expected, in anti‐PD‐L1 treated groups, RASGRP1‐overexpression showed higher levels of CD8^+^ T cells, while no significant difference was found in PBS‐treated groups (Figure 4F‐G). Above results indicated that upregulation of RASGRP1 could enhance the anti‐PD‐L1 response in TNBC.

**FIGURE 4:**
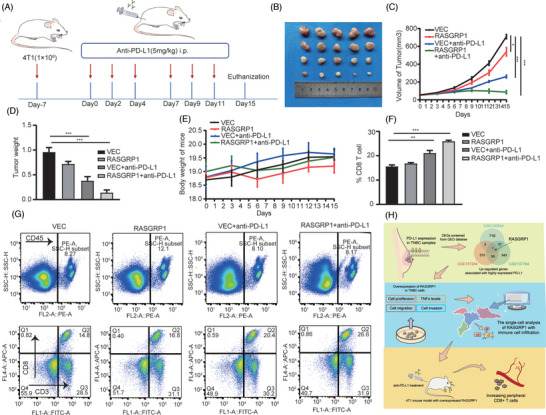
The effects of RAS guanyl releasing protein 1 (RASGRP1) on responses to anti‐programmed death receptor ligand 1 (PD‐L1) treatment in vivo. (A) The protocol of the animal experiments for injection of 4T1 cells and intraperitoneal administration of anti‐PD‐L1 agent in BALB/c mice. (B–D) The size (B), volume (C) and weight (D) of tumours were photographed and measured. (E) The measurement of body weight of BALB/c mice. (F, G) The peripheral proportions of CD8^+^ T cells were detected by flow cytometer and analyzed by Flowjo.10. (H) Schematic diagram showing the biological role of RASGRP1 in immune cell infiltration and anti‐PD‐L1 treatment response.

In all, our study sheds light on the correlation of RASGRP1 expression in tumour cells and infiltrations of CD8^+^ T cells in TNBC TME (Figure 4H). The methods utilized in this work are comprised in Table S3. Previous studies have demonstrated that RASGRP1 involves in mediating immune responses in various diseases. For instance, loss‐of‐function mutations in RASGRP1 impede T‐cell activation and proliferation by interruption of receptor signalling, resulting in lymphoma‐ or leukemogenesis.^11^ In hepatocellular carcinoma, overexpression of RASGRP1 inhibits the inflammation‐associated cancer development, by reducing the production of the proinflammatory cytokine IL‐6.^12^ From now on, the nature of RASGRP1 in TME of TNBC is still blurry. Considering the negative regulation between RASGRP1 and TNF‐α signalling, this effect might be caused by the release of specific cytokines in tumour cells. Our finding suggested that RASGRP1 predicted poor anti‐PD‐L1 agent response and might work as a promising immunotherapy target for anti‐PD‐L1 therapy in TNBC.

## CONFLICT OF INTEREST STATEMENT

The authors declare no conflict of interest.

## Supporting information

Supporting information.
**Figure S1** (A–C) The expression of RASGRP1 was compared between samples with low and high expression of PD‐L1 in GSE100824 (A), GSE107764 (B) and GSE157284 (C) cohorts. (D–F) Expression comparisons of RASGRP1 between negative and positive expression of ER (D), PR (E) and HER2 (F) in GSE162228. (G–I) The expression levels of RASGRP1 in advance Tumor (G), Node (H), and Metastasis (I) using the Xiantao tool. (J–L) The survival curves for the distant metastasis‐free survival (DMFS) (J), overall survival (OS) (K) and recurrence‐free survival (RFS) (L) of BC patients in the high‐ and low‐expression groups of RASGRP1.

Supporting information.
**Figure S2** (A–D) The GSEA enrichment analysis of RASGRP1 through the BEST database. (E) Immune infiltration cells associated with RASGRP1 using ssGESA algorithm from the TCGA database. (F) The correlation between RASGPR1 and immune scores by ESTIMATE algorithm in GSE58812. (G) The heatmap illustrates the relationship between T‐lymphocyte‐related markers and RASGRP1. (H, I) The association of RASGRP1 with CD8B expression in GSE88847 (H) and GSE180775 (I).

Supporting information.
**Figure S3** (A, C) UMAP plots for total cellular components surrounding BC microenvironment in GSE136206 (A) and GSE114727_ inDrop (C). (B, E) Violin plots illustrating RASGRP1 expression in total cellular components based on GSE136206 (B) and GSE114727_ inDrop (E). (D) UMAP plots for RASGRP1 expression in total cellular clusters based on GSE114727_ inDrop. (F, I) UMAP plots showing the major‐lineage cell types in GSE114727_10× (F) and GSE114727_ inDrop (I). (G) UMAP plots unveiling RASGRP1 expression in GSE114727_10×. (H, J) Violin plots revealing RASGRP1 expression in major‐lineage levels in GSE114727_10× (H) and GSE114727_ inDrop (J). (K, M) UMAP plots illustrating the minor‐lineage cell types in GSE114727_ inDrop (K) and GSE114727_10× (M). (L, N) Violin plots showing RASGRP1 expression in minor‐lineage levels in GSE114727_ inDrop (L) and GSE114727_10× (N). (O–Q) The single‐cell analysis for RASGRP1 expression in immune cells of BC microenvironment using GSE195861 from DISCO database. (O) The single‐cell cluster maps show the landscape of immune cell distribution. (P, Q) The cellular components with RASGRP1 expression are illustrated by a cluster map (P) and a bar chart (Q).
Abbreviations: CD4Tconv: conventional CD4^+^T cells; CD8T: CD8^+^ T cells; CD8Tex: Exhausted CD8^+^ T cells; DC: dendritic cell; Mono/Macro: Monocytic cells/ Macrophages; NK: Natural killer cells; Tprolif: proliferative T cells; B: B cells; CD4Tn: naive CD4^+^T cells; CD8Tcm: central memory CD8^+^ T cells; CD8Tem: effector memory CD8^+^ T cells; M1: M1 macrophages; M2: M2 macrophages; cDC2: Type‐2 conventional dendritic cells; pDC: plasmacytoid dendritic cell; CD8Tn: naive CD8^+^T cells; Tfh: T follicular helper cells; Th1: Type 1 T helper cells; Th17: T helper 17 cells.

Supporting information.
**Figure S4** A comparison of TIDE score between high and low expression of RASGRP1 using a TNBC dataset from the TCGA database.

Supporting information.
**Table S1** The DEGs were identified between groups of high and low expression of PD‐L1

Supporting information.
**Table S2** The univariate and multivariate COX analysis of RASGRP1 in BC

Supporting information.
**Table S3** Supplementary for materials and methods
