# Supplementary figures and images for: Targeting RAS guanyl releasing protein 1 promotes T lymphocytes infiltrations and improves anti‐programmed death receptor ligand 1 therapy response of triple‐negative breast cancer
Source: Clin Transl Med. 2023 Jul 17;13(7):e1335. doi: 10.1002/ctm2.1335 (PMC10352603; doi:10.1002/ctm2.1335)

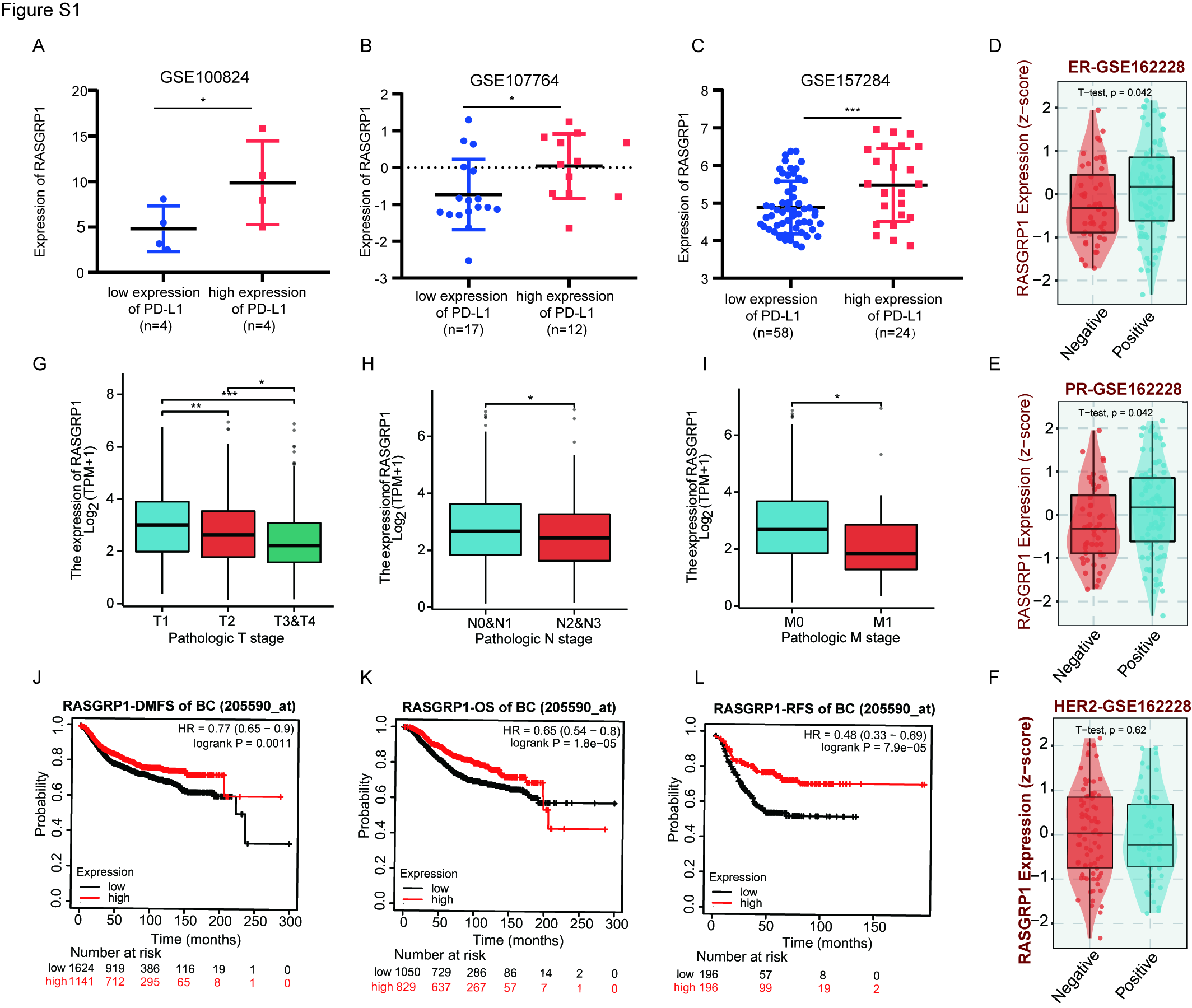

Supplement: Supplementary file 1 — Supporting information. Figure S1 (A–C) The expression of RASGRP1 was compared between samples with low and high expression of PD‐L1 in GSE100824 (A), GSE107764 (B) and GSE157284 (C) cohorts. (D–F) Expression comparisons of RASGRP1 between negative and positive expression of ER (D), PR (E) and HER2 (F) in GSE162228. (G–I) The expression levels of RASGRP1 in advance Tumor (G), Node (H), and Metastasis (I) using the Xiantao tool. (J–L) The survival curves for the distant metastasis‐free survival (DMFS) (J), overall survival (OS) (K) and recurrence‐free survival (RFS) (L) of BC patients in the high‐ and low‐expression groups of RASGRP1. [file CTM2-13-e1335-s007.tif]

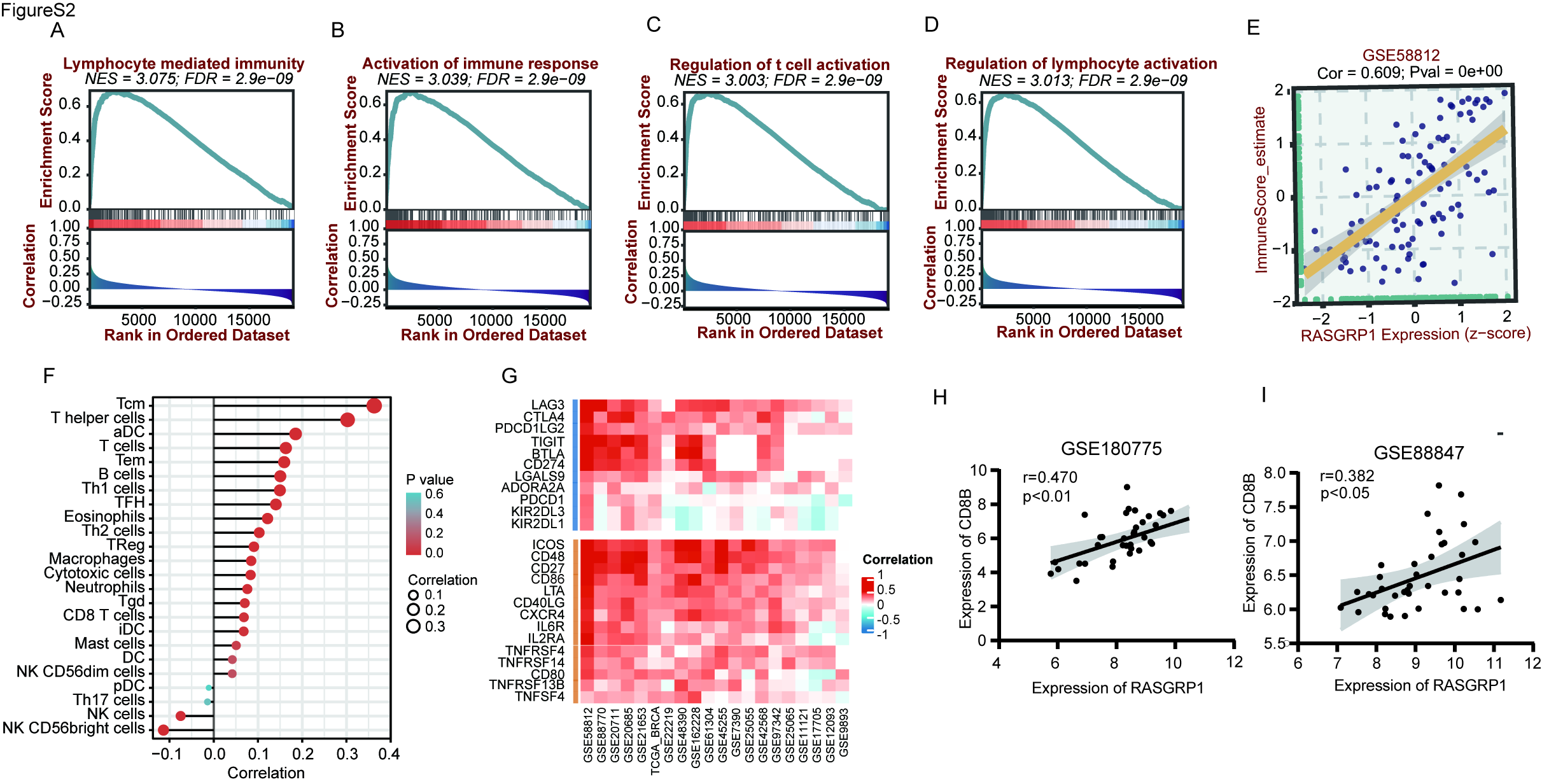

Supplement: Supplementary file 2 — Supporting information. Figure S2 (A–D) The GSEA enrichment analysis of RASGRP1 through the BEST database. (E) Immune infiltration cells associated with RASGRP1 using ssGESA algorithm from the TCGA database. (F) The correlation between RASGPR1 and immune scores by ESTIMATE algorithm in GSE58812. (G) The heatmap illustrates the relationship between T‐lymphocyte‐related markers and RASGRP1. (H, I) The association of RASGRP1 with CD8B expression in GSE88847 (H) and GSE180775 (I). [file CTM2-13-e1335-s006.tif]

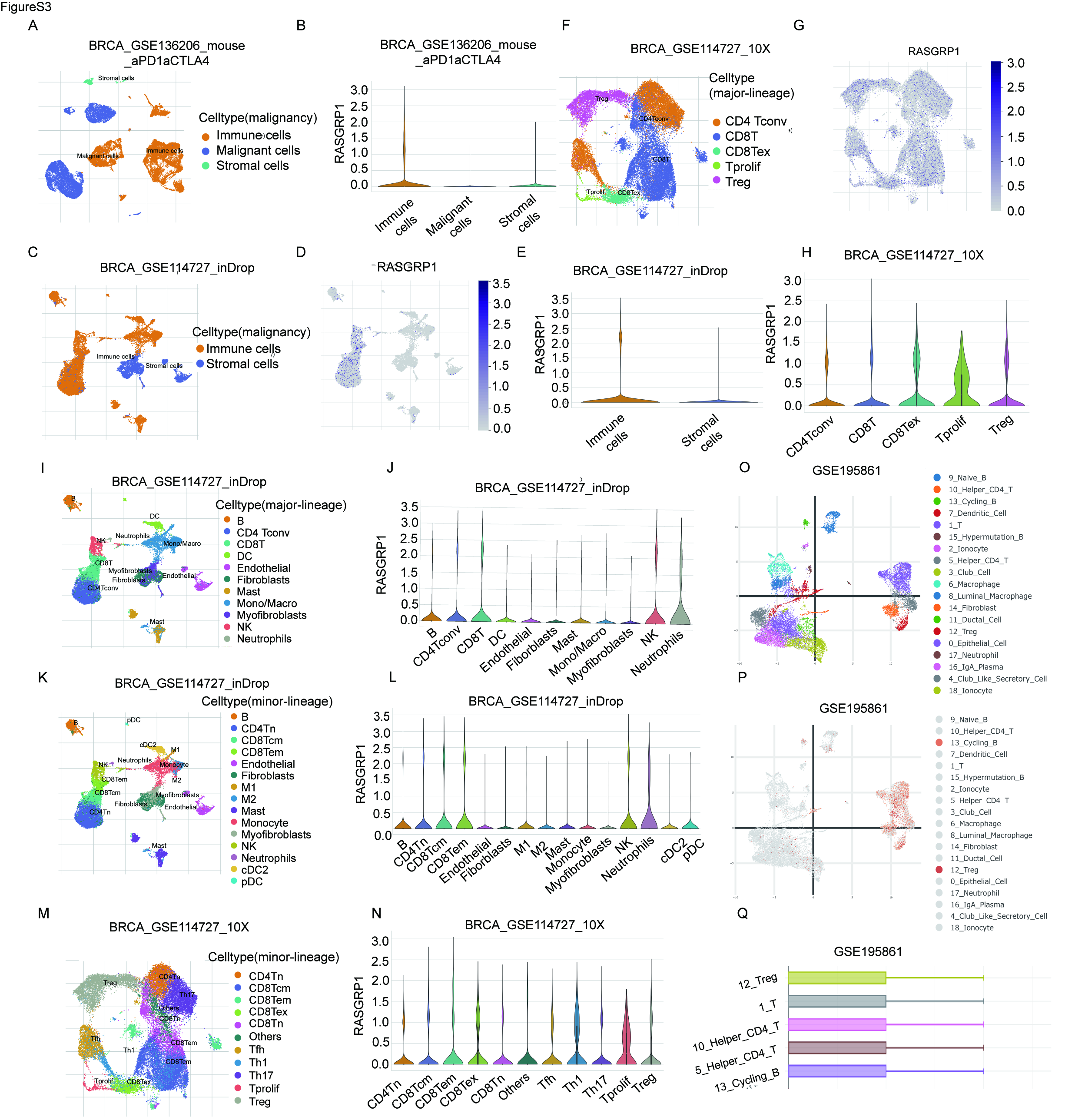

Supplement: Supplementary file 3 — Supporting information. Figure S3 (A, C) UMAP plots for total cellular components surrounding BC microenvironment in GSE136206 (A) and GSE114727_ inDrop (C). (B, E) Violin plots illustrating RASGRP1 expression in total cellular components based on GSE136206 (B) and GSE114727_ inDrop (E). (D) UMAP plots for RASGRP1 expression in total cellular clusters based on GSE114727_ inDrop. (F, I) UMAP plots showing the major‐lineage cell types in GSE114727_10× (F) and GSE114727_ inDrop (I). (G) UMAP plots unveiling RASGRP1 expression in GSE114727_10×. (H, J) Violin plots revealing RASGRP1 expression in major‐lineage levels in GSE114727_10× (H) and GSE114727_ inDrop (J). (K, M) UMAP plots illustrating the minor‐lineage cell types in GSE114727_ inDrop (K) and GSE114727_10× (M). (L, N) Violin plots showing RASGRP1 expression in minor‐lineage levels in GSE114727_ inDrop (L) and GSE114727_10× (N). (O–Q) The single‐cell analysis for RASGRP1 expression in immune cells of BC microenvironment using GSE195861 from DISCO database. (O) The single‐cell cluster maps show the landscape of immune cell distribution. (P, Q) The cellular components with RASGRP1 expression are illustrated by a cluster map (P) and a bar chart (Q). Abbreviations: CD4Tconv: conventional CD4+T cells; CD8T: CD8+ T cells; CD8Tex: Exhausted CD8+ T cells; DC: dendritic cell; Mono/Macro: Monocytic cells/ Macrophages; NK: Natural killer cells; Tprolif: proliferative T cells; B: B cells; CD4Tn: naive CD4+T cells; CD8Tcm: central memory CD8+ T cells; CD8Tem: effector memory CD8+ T cells; M1: M1 macrophages; M2: M2 macrophages; cDC2: Type‐2 conventional dendritic cells; pDC: plasmacytoid dendritic cell; CD8Tn: naive CD8+T cells; Tfh: T follicular helper cells; Th1: Type 1 T helper cells; Th17: T helper 17 cells. [file CTM2-13-e1335-s002.tif]

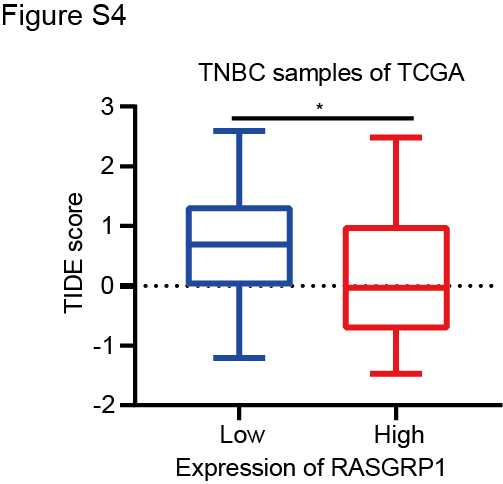

Supplement: Supplementary file 4 — Supporting information. Figure S4 A comparison of TIDE score between high and low expression of RASGRP1 using a TNBC dataset from the TCGA database. [file CTM2-13-e1335-s005.jpg]
