# Supplementary material for: Targeting RAS guanyl releasing protein 1 promotes T lymphocytes infiltrations and improves anti‐programmed death receptor ligand 1 therapy response of triple‐negative breast cancer
Source: Clin Transl Med. 2023 Jul 17;13(7):e1335. doi: 10.1002/ctm2.1335 (PMC10352603; doi:10.1002/ctm2.1335)
Supplement: Supplementary file 6 — Supporting information. Table S2 The univariate and multivariate COX analysis of RASGRP1 in BC [file CTM2-13-e1335-s001.docx]

**Table S2 The univariate and multivariate COX analysis of RASGRP1 in BC**

| Characteristics | Total(N) | HR(95% CI) Univariate analysis | P value Univariate analysis | HR (95% CI) Multivariate analysis | P value Multivariate analysis |
| --- | --- | --- | --- | --- | --- |
| T stage | 1079 |  |  |  |  |
| T1 | 276 | Reference |  |  |  |
| T2&T3&T4 | 803 | 1.482 (1.007-2.182) | 0.046 | 1.050 (0.540-2.040) | 0.887 |
| N stage | 1063 |  |  |  |  |
| N0 | 514 | Reference |  |  |  |
| N1&N2&N3 | 549 | 2.239 (1.567-3.199) | <0.001 | 1.992 (1.253-3.168) | 0.004 |
| M stage | 922 |  |  |  |  |
| M0 | 902 | Reference |  |  |  |
| M1 | 20 | 4.254 (2.468-7.334) | <0.001 | 2.865 (1.436-5.716) | 0.003 |
| Age | 1082 |  |  |  |  |
| <=60 | 601 | Reference |  |  |  |
| >60 | 481 | 2.020 (1.465-2.784) | <0.001 | 2.328 (1.578-3.436) | <0.001 |
| PR status | 1029 |  |  |  |  |
| Positive | 687 | Reference |  |  |  |
| Negative | 342 | 1.367 (0.977-1.912) | 0.068 | 1.232 (0.683-2.225) | 0.488 |
| ER status | 1034 |  |  |  |  |
| Positive | 792 | Reference |  |  |  |
| Indeterminate | 2 | 18.392 (4.478-75.532) | <0.001 | 8.748 (1.112-68.802) | 0.039 |
| Negative | 240 | 1.405 (0.978-2.020) | 0.066 | 1.811 (0.970-3.381) | 0.062 |
| HER2 status | 727 |  |  |  |  |
| Positive | 157 | Reference |  |  |  |
| Indeterminate | 12 | 0.000 (0.000-Inf) | 0.994 |  |  |
| Negative | 558 | 0.628 (0.383-1.028) | 0.064 |  |  |
| Pathologic stage | 1059 |  |  |  |  |
| Stage I | 180 | Reference |  |  |  |
| Stage II&Stage III&Stage IV | 879 | 2.210 (1.313-3.721) | 0.003 | 1.218 (0.489-3.034) | 0.672 |
| Race | 993 |  |  |  |  |
| Asian | 60 | Reference |  |  |  |
| Black or African American | 180 | 1.525 (0.463-5.024) | 0.488 |  |  |
| White | 753 | 1.325 (0.420-4.186) | 0.631 |  |  |
| RASGRP1 | 1082 |  |  |  |  |
| Low | 540 | Reference |  |  |  |
| High | 542 | 0.661 (0.480-0.910) | 0.011 | 0.589 (0.403-0.861) | 0.006 |
