# Supplementary material for: Targeting RAS guanyl releasing protein 1 promotes T lymphocytes infiltrations and improves anti‐programmed death receptor ligand 1 therapy response of triple‐negative breast cancer
Source: Clin Transl Med. 2023 Jul 17;13(7):e1335. doi: 10.1002/ctm2.1335 (PMC10352603; doi:10.1002/ctm2.1335)
Supplement: Supplementary file 7 — Supporting information. Table S3 Supplementary for materials and methods [file CTM2-13-e1335-s003.docx]

**MATERIALS AND METHODS**

**1 Bioinformatics Analyses**

The GEO (http://www.ncbi.nlm.nih.gov/geo) is an open-source platform for the storage of high throughput gene expression data, chips, and microarrays data ^1^. Three expression profiling datasets (GSE100824, GSE157284 and GSE107764) were downloaded from the GEO database, respectively. The GSE100824 dataset includes MDA-MB-231 breast cancer cells with PD-L1 overexpression plasmid or empty plasmid. The GSE107764 dataset includes TNBC samples with high (N=12) or low (N=17) levels of PD-L1 expression. The GSE157284 dataset includes 58 PD-L1-negative and 24 PD-L1-postive TNBC cases, in which immunohistochemistry (IHC) score≥4 defined as positive expression of PD-L1, and <4 considered as negative PD-L1 expression. According to the setting cut-off criteria (Foldchange>1.5, P<0.05), the DEGs between high and low expression of PD-L1 were identified in TNBC cells and tissues. Furthermore, the association between RASGRP1 and PD-L1 expression was identified by spearman correlation analysis in GSE88847 and GSE180775, and also was verified in GSE55812 using the Biomarker Exploration of Solid Tumors database (BEST, <https://rookieutopia.com/app_direct/BEST/>)^2^.

The single cell analysis was performed using the TISCH (http:// tisch. comp-genom ics. org/home/) and DISCO database (https://www.immunesinglecell.org/). TISCH, a resource of single-cell RNA-seq data, contains detailed cell-type annotation of tumor microenvironment (TME) from tissues of distinct species, enabling visualization of various cells distribution ^3^. DISCO, an open platform, collects a comprehensive collection single-cell RNA-seq datasets, and efficiently performs integrated analysis in a wide range of tissues and diseases ^4^. The Seurat version 4.3.0 R toolkit was used for analyzing the single-cell RNAseq using EMTAB8107 and GSE136206.

The ER-, PR- and Her2-negative or positive BC samples were implemented to compare the RASGRP1 expression in GSE162228 by BEST database. To further examine the prognostic impacts of RASGRP1 on prognosis, Kaplan-Meier plotter (https://kmplot.com/analysis/) was exploited to analysis prognostic index including the DMFS, OS and RFS in BC and TNBC patients. Furthermore, we compared RASGRP1 levels in BC patients at TNM stages through the Xiantao tool (<https://www.xiantao.love/>) ^5^. To detect whether RASGRP1 could predict prognosis as an independent factor in BC patients, we utilized the Xiantao tool to perform nomgram model, univariate, and multivariate Cox regression analysis.

To determine the RASGRP1-related signal pathways, KEGG and GO analysis was performed through BEST database. The enrichment of hallmark gene sets signaling was conducted by TISCH database. Furthermore, tumor-infiltrating immune cells linked with RAGRRP1 expression were examined by BEST database and Xiantao tool, respectively. Based on BEST database, 20 BC GEO datasets, in which contains 2 TNBC datasets (GSE55812 and GSE97342), were extracted for correlation analysis. In detail, the ESTIMATE, MCP counter and TIMER algorithm were applied to estimate the correlations between RASGRP1 and immunocytes. We further used Xiantao tool to verify the relationship between RASGRP1 and distinct immune cells in TCGA datasets of BC by ssGSEA algorithm. Furthermore, we reaffirmed the links between RASGRP1 and CD8^+^T cells utilizing the ESTIMATE and XCELL algorithm by BEST database using TNBC dataset GSE55812.

Moreover, we also used BEST database to visualize the associations between RASGRP1 and T- lymphocyte-related markers in GEO datasets of BC. Additionally, we assessed the links of RASGRP1 with CD8A and CD8B in GSE88847 and GSE180775. An ICB response was predicted employing Tumor Immune Dysfunction and Exclusion (TIDE) algorithm using TCGA dataset of TNBC.

**2 Cell Lines and Reagents**

Human (BT549 and MDA-MB-231) and murine (4T1) TNBC cell lines were obtained from the Center for Molecular Medicine at Xiangya Hospital. BT549 cell was cultured using 1640 medium (8122374, Gibco™, United States), while 4T1 and MDA-MB-231 were placed in Dulbecco’s modified eagle medium (DMEM. Gibco, Billings, MT, USA) with 10% fetal bovine serum (Gibco, USA) and 1% penicillin-streptomycin (Gibco, USA). Anti-PD-L1 antibody (HY-P9904) was purchased from MedChemExpress (NJ, USA).

**3 Transfection and lentiviral transduction**

The overexpression plasmid RASGRP1-pcDNA3.1 was purchased from Sangon Biotech (Guangzhou, China). According to the manufacturer’s instructions, RASGRP1-pcDNA3.1 and empty pcDNA3.1(+) plasmid as control were transfected, separately, into BT549 and MDA-MB-231 cells for 24 h using lipofectamine 3000 (L300015, Thermo Fisher Scientific, USA). The stable overexpression plasmid Rasgrp1-GV348 and relative negative control plasmids were purchased from GeneChem (Shanghai, China). 4T1 cells were infected by lentivirus solutions for 48h, and cells with the stably overexpressed RASGRP1 were selected using puromycin (10 μg/ml, Sangon Biotech, A610593) for a total of 10 days. The protein expression of RASGRP1 was detected via Western Blotting.

**4 Cell counting kit 8 assay**

For CCK-8 assay, BT549 and MDA-MB-231 cells were seeded in 96 well culture plates (2×10^3^ cells/well) after cells transfected with pcDNA3.1-RASGRP1 or vector for 24h. Then, CCK-8 test solution (Bimake, Houston, TX, USA) was used to detect cell proliferation at 24h, 48h, 72h, 96h, and 120h. Cell viability was assessed by a VICTOR X2 microplate reader (PerkinElmer, Waltham, MA, USA) at a test wavelength of 450 nm.

**5 Colony formation assay**

After transfection for 24h, BT549 and MDA-MB-231 cells were seeded in 6-well plates (1×10^3^ cells/well). Cells were incubated at 37°C for approximately 2 weeks, and then washed twice with PBS and stained with 0.3% w/v crystal violet/methanol for 20 min at room temperature.

**6 Transwell and wounding healing assays**

The transwell assay was performed using 24-well transwell plates (8.0 μm pore size; Corning, USA) and Matrigel (BD, USA). After transfection for 24h, BT549 and MDA-MB-231 cells (1×10^5^/200ul) were digested by trypsin and re-suspended in serum-free media. The cells were placed into the upper compartment, and then 600ul DMEM containing 10% FBS was added into the bottom chamber. After incubation for 24 h, invasion cells were fixated utilizing 4% paraformaldehyde for 30 min, and stained with 0.1% crystal violet. Invasive cells were captured by inverted microscope within 5 randomly selected fields, and counted by image J.

For the wound-healing migration assay, cells were seeded in 6-well plates after transfection for 24 h. The cells were scratched by a 200 μL pipette tip, and then washed with PBS and incubated at serum-free media. Wounds were observed under a microscope and photographed at 0 and 24h. The wound-healing migration area was measured and analyzed by Image J.

**7 Western Blot assay**

Cells were lysed in a RIPA buffer supplemented with protease inhibitor cocktail (Thermo, Rockford, USA). Protein extracts were separated via electrophoresis on 10% SDS–PAGE and transferred to PVDF membranes. The membranes were blocked utilizing 5% skimmed milk, and then incubated with anti-RASGRP1(1:1000, A10495, ABclonal, China) and β-actin (1:2000; sc-58673, Santa, USA) antibodies at 4 °C overnight. Next, the membranes were incubated with the HRP-conjugated secondary antibodies (Cat NO: SA00001-1, Proteintech, USA) for 1 h at room temperature. The bands were visualized using an enhanced chemiluminescence detection kit (34580, Thermo Scientific, USA) through the Image Lab software (Bio-Rad, USA).

**8 ELISA assays for TNF-α detection**

The ELISA assays kits for examining human and murine TNF-α levels were purchased from Sangon Biotech (D711045; D721217). According to the manufacturer’s instructions, 1×10^6^ cells were lysis by sonication, and supernatants were collected after centrifuging at 1500 g for 10 min at 4°C. Then, 100 μL/well of supernatants was added in ELISA plate, and was incubated for 1.5 h at 37°C. 100 μL/well biotin-conjugated antibody (1:100), and streptavidin-HRP (1:100) in reagent diluent was added in sequence to each well for incubation. 90 μL/well substrate solution was then added to each well in dark, and the reaction was stopped by adding 50 μL of stop solution. The OD value of each well was detected using the microplate reade at wavelength of 450 nm.

**9 IHC**

The 21 human TNBC tissues were obtained from the Department of Pathology, Xiangya Hospital. The studies involving human participants were reviewed and approved by the ethics committee of Xiangya Hospital, Central South University (202303035). The tissues were paraffin embedded, and IHC scoring was performed as described previously ^6^. The samples were incubated with the primary antibody against RASGRP1(1:500, A10495, ABclonal, China) and CD8 (1:500, 66868-1-Ig, Proteintech, USA). Two pathologists examined and differentially quantified the images of the sections. According to their total scores, the evaluation of IHC intensity was performed and a score of 0 (negative), 1 (weak brown), 2 (moderate brown), or 3 (strong brown) was assigned, while the extent of staining was evaluated by assigning scores of 0 (≤10%), 1(11%–25%), 2 (26%–50%), 3 (51%–75%), or 4 (>75%). The final staining score was determined by multiplying the intensity score and extent score and classified as weakly positive (1–3), positive (4–6), and strongly positive (7–12). All paraffin-embedded specimens were collected in accordance with the ethical standards of the human experimental committee.

**10 *In vivo* mouse model**

20 six-week-old female Balb/c mice were purchased from the Department of Laboratory Animals of Central South University. Animal studies were approved by the Department of Laboratory Animals of Central South University (CSU-2022-0644). For the subcutaneous tumor model, 4T1 cells with stable overexpression of RASGRP1 or empty vector were injected into the mammary fat pad of BALB/C mice (5× 10^5^/mouse). When the tumor volume reached ~ 50 mm^3^, mice were randomly assigned into 4 groups according to mean tumor volume (n=5). The anti-PD-L1 antibody (5mg/kg) or PBS was intraperitoneally administered every 2 days for a total of 6 times to the mice. Tumor size was measured by a caliper every 3 days and was calculated by using the following formula: Volume = 1/2 × (length × width^2^). After 15 days of cell inoculation, EDTA-anticoagulated whole blood was collected, and the mice were euthanized for assessing tumor.

**11 Flow cytometry**

Cells extracted from peripheral blood were treated with red blood cell lysis buffer for 5min. Next, cell suspensions were centrifuged at 400 g for 5 min at 4°C. After discarding the supernatants, cell pellets were incubated with anti-mouse CD45-PE antibody (12-0451-82, ebioscience, USA), FITC-CD3 antibody (100203, biolegend, USA) and CD8a-APC antibody (100712, biolegend, USA), on ice, in the dark, for 20 min. The cell pellets were re-suspended in 400 μL of flow buffer and filtered through a 100 μm mesh. The Samples were then tested using a flow cytometer, and data were analyzed using FlowJo V10 software (FlowJo, LLC). The CD8^+^ T cells were defined as follows: CD45^+^ cells were defined as leukocyte population, and the CD8^+^ T cells were then identified based on positive expression of CD3 and CD8.

**12 Statistical analyses**

All experiments were examined at least in triplicated times. To perform differences comparison based on different groups, 2 data groups was compared by Student’s t-test, and ANOVA were conducted for more than 2 data groups. Univariate and multivariate Cox regression was performed for survival analysis. Data analysis was performed using statistical software, GraphPad Prism 8 and SPSS 23.0. Significant differences were considered at *, P<0.05; **, P<0.01; ***, P<0.001. for all tests.

**Reference**

1. Barrett T, Wilhite SE, Ledoux P, et al. NCBI GEO: archive for functional genomics data sets--update. *Nucleic Acids Res*. Jan 2013;41(Database issue):D991-5. doi:10.1093/nar/gks1193

2. Liang Q, Xu Z, Liu Y, et al. NR2F1 Regulates TGF-beta1-Mediated Epithelial-Mesenchymal Transition Affecting Platinum Sensitivity and Immune Response in Ovarian Cancer. *Cancers (Basel)*. Sep 24 2022;14(19)doi:10.3390/cancers14194639

3. Sun D, Wang J, Han Y, et al. TISCH: a comprehensive web resource enabling interactive single-cell transcriptome visualization of tumor microenvironment. *Nucleic Acids Res*. Jan 8 2021;49(D1):D1420-D1430. doi:10.1093/nar/gkaa1020

4. Li M, Zhang X, Ang KS, et al. DISCO: a database of Deeply Integrated human Single-Cell Omics data. *Nucleic Acids Res*. Jan 7 2022;50(D1):D596-D602. doi:10.1093/nar/gkab1020

5. Yan Y, Liang Q, Xu Z, Yi Q. Integrative bioinformatics and experimental analysis revealed down-regulated CDC42EP3 as a novel prognostic target for ovarian cancer and its roles in immune infiltration. *PeerJ*. 2021;9:e12171. doi:10.7717/peerj.12171

6. Choudhury KR, Yagle KJ, Swanson PE, Krohn KA, Rajendran JG. A robust automated measure of average antibody staining in immunohistochemistry images. *J Histochem Cytochem*. Feb 2010;58(2):95-107. doi:10.1369/jhc.2009.953554
